# Does preoperative opioid therapy in patients with a single lumbar disc herniation positively influence the postoperative outcome detected by quantitative sensory testing?

**DOI:** 10.1007/s10143-022-01818-z

**Published:** 2022-05-24

**Authors:** Lea Gasser, Sara Lener, Sebastian Hartmann, Wolfgang N. Löscher, Claudius Thomé, Anja Hofer

**Affiliations:** 1 Department of Neurosurgery, Medical University of Innsbruck, Innsbruck, Austria; 2 Department of Neurology, Medical University of Innsbruck, Innsbruck, Austria

**Keywords:** Lumbar sequesterectomy, Quantitative sensory testing, Opioid therapy, Lumbar disc herniation, Lumbar radiculopathy

## Abstract

The importance of the type of pain medication in spinal disease is an ongoing matter of debate. Recent guidelines recommend acetaminophen and NSAIDs as first-line medication for lumbar disc herniation. However, opioid pain medication is commonly used in patients with chronic pain, and therefore also in patients with sciatica. The aim of this study is to evaluate if opioids have an impact on the outcome in patients suffering from lumbar disc herniation. To assess this objectively quantitative sensory testing (QST) was applied. In total, 52 patients with a single lumbar disc herniation confirmed on magnetic resonance imaging (MRI) and treated by lumbar sequesterectomy were included in the trial. Patients were analysed according to their preoperative opioid intake: 35 patients who did not receive opioids (group NO) and 17 patients, who received opioids preoperatively (group O). Further evaluation included detailed medical history, physical examination, various questionnaires, and QST. No pre- and postoperative differences were detected in thermal or mechanical thresholds (*p* > 0.05). Wind-up ratio (WUR) differed significantly between groups 1 week postoperatively (*p* = 0.025). The NRS for low back pain was rated significantly higher in the non-opioid group (NO) after 1-week follow-up (*p* = 0.026). Radicular pain tended to be higher in the NO group after 12 months of follow-up (*p* = 0.023). Opioids seem to be a positive predictor for the postoperative pain outcome in early follow-up in patients undergoing lumbar sequesterectomy. Furthermore, patients presented with less radicular pain 1 year after surgery.

## Introduction

Low back pain (LBP) is often caused by degenerative spinal disease, like lumbar disc herniations. Due to its high prevalence, LBP is one of the most common and challenging pain conditions encountered in clinical practice and is often accompanied by radiculopathy [11, 20]. Furthermore, it poses an immense socioeconomic burden by making a major contribution to healthcare expenses [10]. Over the past few years, surgeries and hospitalizations due to herniated discs showed increasing numbers. Patients, suffering from lumbar disc herniation and undergoing surgery experience significant improvements in pain and quality of life postoperatively [2, 23, 27, 45]. However, prior to surgery, guidelines advocate medical treatment with pain killers as first-line therapy. Despite recent guidelines recommend acetaminophen and non-steroidal anti-inflammatory drugs (NSAIDs) as first-line pharmacologic pain treatment, a great number of affected patients use opioid pain medications to alleviate pain [7, 12, 22, 43]. Due to the high incidence and complexity of LBP treatment, with or without radiculopathy, patients are more likely to receive high doses of opioids compared to patients with other pain diagnoses [4, 10, 21]. This occurs, even though opioids show various side effects and bear serious long-term effects [17, 29]. The frequent prescription of opioids might also be caused by the fact that opioids have proven to reduce not only nociceptive, but also neuropathic pain components, which might both be present in LBP patients[37]. Nevertheless, evidence for their long-term impact on pain relief, functional capacity, and health-related quality of life is still controversial and requires further research [8, 39, 44]. Considering the potential effect of opioids on altered pain sensitivity, the aim of this study was to examine whether preoperative opioid pain medication in patients undergoing lumbar sequesterectomy influences the postoperative outcome in general detected by quantitative sensory testing (QST) specifically.

## Material and methods

### Subjects

The study was purely observational, and pain management was not delayed or altered by participation in this study. Participants were recruited between 2014 and 2018. All subjects gave their informed consent. The study was approved by the local ethics committee (Medical University of Innsbruck) in accordance with the ethical principles originating from the Declaration of Helsinki and in compliance with Good Clinical Practice. Patients were considered for inclusion if they were aged between 18 and 65 years and had a single-level disc herniation confirmed on magnetic resonance imaging (MRI) and medically refractory radicular pain and/or motor deficits in the corresponding nerve root distribution of L3 to S1. Surgery was indicated if pain was unresponsive to nonoperative treatment for at least 6 weeks or if signs of severe nerve root compression, such as motor weakness, hypoesthesia, saddle anaesthesia, dysfunction of bladder, and/or bowel were present. All patients had an indication for sequesterectomy according to the guidelines of the German Society of Neurosurgery (DGNC) and the German Society of Orthopedics and Orthopedic Surgery (DGOOC). The exclusion criteria are listed in detail in Table 1. Preoperative pain medication was prescribed by general or private practitioners and administered orally. After admission to the hospital, oral medication was continued and extended to subcutaneous administration of opioids occasionally. Postoperative pain management was performed according to the institutions standard of care including the admission of piritramid subcutaneously on an occasional base on within the first 24 h after surgery and continuing the oral administration NSAIDs as well as metamizole or acetaminophen for 5–10 days postoperatively, regarding the patient’s subjective assessment.

**Table 1:** Exclusion criteria

Exclusion criteria
Previous surgery at index level that caused a sensory nerve dysfunction Any degenerative muscular or neurological condition that would interfere with evaluation of outcome measures including but not limited to Parkinson’s disease, amyotrophic lateral sclerosis, multiple sclerosis, muscular dystrophy, and myelopathic diseases of different causes Participation in a clinical trial within the last 3 months Unable or unwilling to comply therapeutic instructions or to comply the follow-up visits at the study site Active or chronic infection, systemic or local, systemic disease including HIV, AIDS, hepatitis Active malignancy defined as a history of any invasive malignancy, except nonmelanoma skin cancer, unless the patient has been treated with curative intent and there have been no clinical unable to undergo MRI Neoplasia as the source of symptoms, diabetes mellitus Paget’s disease, osteomalacia, or any other metabolic bone disease. skin disease that influences sensory nerve function Polyneuropathy Autoimmune disorder that impacts the musculoskeletal system (i.e. lupus, rheumatoid arthritis, ankylosing spondylitis) Acute episode or major mental illness (psychosis, major affective disorder, or schizophrenia) and usage of antidepressive drugs Physical symptoms without a diagnosable medical condition to account for the symptoms, which may indicate symptoms of psychological rather than physical origin Recent or current history of substance abuse (drugs, alcohol, narcotics, recreational drugs). Pursuing personal litigation related to spinal diseases Prisoner or ward of the state

### Questionnaires, medical history, and clinical examination

The prospectively planned evaluation included detailed medical history, physical examination, and various questionnaires. The numeric rating scale (NRS) was determined separately for back and leg pain. Higher scores on a 0 to 10 rating scale indicate worse pain (0 = no pain, 10 = most intense pain imaginable) [19]. The Beck depression inventory (BDI) was applied in all patients, to measure the severity of depression and responsiveness to treatment [40]. To identify neuropathic pain components, the painDETECT questionnaire (PD-Q) was performed. Patients, who score ≤ 12, are unlikely to have a neuropathic pain component, whereas patients who score ≥ 19 are very likely to suffer from neuropathic pain [15, 16]. The degree of pain-related disability and the therapeutic effect were assessed with the Oswestry disability index (ODI), which is a widely used condition-specific outcome measure in patients with spinal disorders. It is divided into ten sections designed to assess multiple aspects of disability with respect to pain. The outcome score is defined as follows: 0–20% indicates minimal disability, 20–40% moderate disability, 40–60% severe disability, 60–80% very serious disability, and 80–100% bed-bound disability [11]. The Core outcome measure index (COMI) is used to monitor the outcome of spinal surgery from the patient’s perspective. It comprises a series of questions covering the domains of back and leg pain intensity, back specific function, symptom-specific well-being, general quality of life, work disability and social disability, global effectiveness of surgery, and patient’s satisfaction with treatment [22, 24]. Furthermore, the EuroQol-5Dimension (EQ-5D) questionnaire and thermometer were used to assess the quality-adjusted health status [5]. Additionally, the neurological status and the quality and quantity of current pain medication in accordance to the WHO guidelines for pain treatment, including nerve root and facet joint injections, were documented. All data were recorded the day before surgery, within 1 week, and 6 and 12 months after surgery.

### Quantitative sensory testing

The quantitative sensory testing (QST) is a standardized computer-controlled method and is currently the only available technique to quantitatively assess the functional state of the somatosensory system [31]. QST allows to evaluate the function of unmyelinated C-fibres, thinly myelinated A-delta fibres, and thickly myelinated A-beta fibres and their projection pathways using seven individual tests to assess 13 individual parameters. QST was performed pre- and postoperatively by a single investigator according to the official testing protocol [36]. Thermal tests were performed using a Sensory Analyzer TSA-II (Medoc, Ramat Yishai, Israel). Cold and warm detection thresholds were measured first (CDT, WDT), followed by the measurement of cold pain and heat pain thresholds (CPT, HPT). The mechanical detection threshold (MDT) was measured with a standardized set of modified von Frey hairs (Somedic, Sösdala, Sweden) that exert forces upon bending between 0.25 and 512 mN. The vibration detection threshold (VDT) was performed with a Rydel-Seiffer tuning fork (64 Hz, 8/8 scale). The mechanical pain threshold (MPT) was measured by a custom-made pinprick set with forces from 8 to 512 mN. Mechanical pain sensitivity (MPS) was assessed using the same pinprick stimuli to obtain a stimulus response function for pinprick evoked pain. Subjects were asked to give a pain rating for each stimulus on a 0 to 10 NRS. A pressure gauge device (FDK 20, Wagner Instruments, Greenwich, CT, USA) was used to measure the pressure pain threshold (PPT). The wind-up ratio (WUR) is described as the increase in pain intensity over time, when a given stimulus is repeated above a critical rate. It is caused by repeated stimulation of group C peripheral nerve fibres, leading to increasing electrical response in the corresponding posterior horn neurons and is represented by comparing the perceived intensity of a single pinprick stimulus (256 mN) to a train of 10 pinprick stimuli of the same force applied at a 1/s rate. Subjects rated the experienced pain of the single stimulus and thereafter the pain at the end of the test by using a numerical rating scale. The pain rating of the 10 repetitive pinprick stimuli was then divided by the pain rating to a single stimulus to calculate the WUR [35, 36].

### Surgical procedures

Surgery was performed in a standardized manner by two surgeons after induction of general endotracheal anaesthesia and with the assistance of an operating microscope (Pentero, Carl Zeiss Co., Wetzlar, Germany) while the patient was in a prone position. The spinal canal was exposed by performing a minimal interlaminar fenestration in cases of non-dislocated or caudally herniated discs. In cases of cranially herniated discs, a translaminar approach was undertaken. Based on results of previous trials, only the herniated material was removed, and the herniated space was not entered, if at all possible [42]. Medial facetectomy was not necessary in any operative case. Intraoperative problems such as surgery related complications and postoperative complications like reoperations, recurrent disc herniations, infection, or hematoma were recorded. All patients were treated by our institutional standard of care regarding anaesthesia and received 0.2–0.4 mg fentanyl for induction of anaesthesia, as well as 7.5–15 mg of piritramid before emergence. Continuous anaesthesia was inducted by propofol and remifentanil administered intravenously. None of the patients received local anaesthetics before or after wound closure.

### Statistical analysis

All patients with a complete preliminary examination were considered for inclusion into the analysis. All values were expressed as mean ± SD. The Kolmogorov–Smirnov test was used for testing normal distribution. The unpaired student’s *t* test, Mann–Whitney *U* test, and Fisher’s exact test were used to analyse differences in clinical and demographic characteristics and in clinical outcome variables. A *p* value < 0.05 was considered statistically significant. All statistical evaluations were performed with SPSS Version 21.0 (IBM Corp. Released 2012. IBM SPSS Statistics for Windows, Version 21.0, NY: IBM Corp.). Figures were designed Microsoft Office 365 Excel (version 18.1910.1283.0, Microsoft, Redmond, Washington, USA).

## Results

**Fig. 1:**
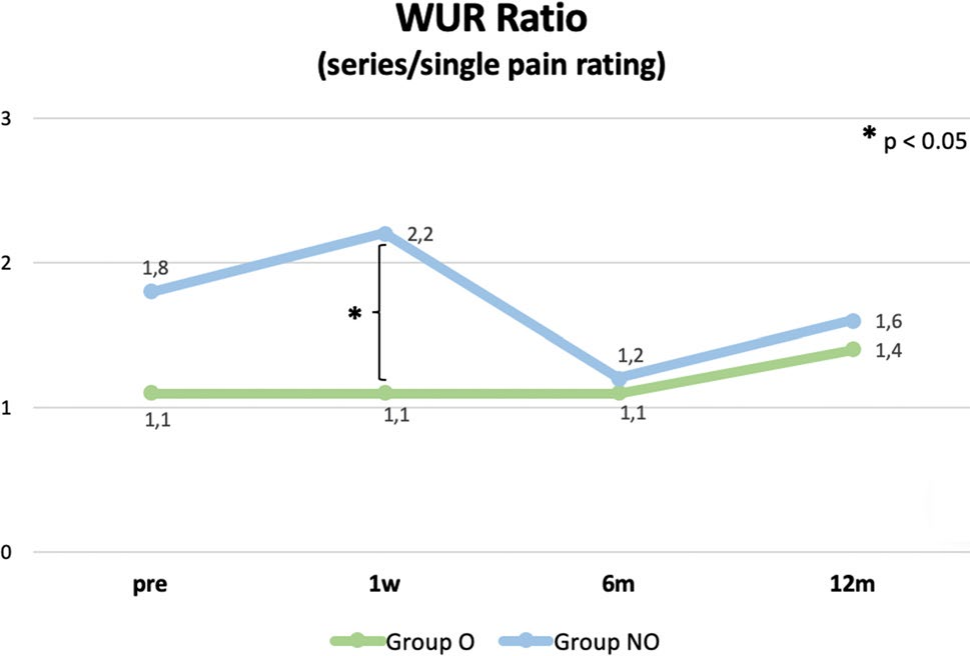
Wind-up ratio (WUR) for group O and group NO preoperatively, 1 week, 6 months, and 12 months postoperatively

A total of 52 consecutive patients met the initial inclusion criteria and were enrolled in the clinical trial. By chance, the cohort was divided into patients with regular opioid intake (group O; *n* = 17) and those without (group NO; *n* = 35). There were no significant intergroup differences in the preoperative demographic data (Table 2). All patients (*n* = 52) received non-opioid medication preoperatively (Table 3) whereas 3 patients (6%) received gabapentin, 1 patient (2%) received pregabalin, 20 patients (38%) received NSAIDs (including naproxen, diclofenac, and dexibuprofen), 7 patients (13%) received metamizole, and 8 patients (15%) received paracetamol/acetaminophen (Table 3). All mentioned drugs were administered orally. In the patient group receiving opioids, 10 patients (19%) were prescribed tramadol, 3 patients (6%) received oxycodone, and 6 patients (12%) were treated with piritramid (Table 3). Tramadol and oxycodone were administered orally whereas piritramid was administered subcutaneously. None of the patients developed pharmacological adverse events. The mean duration of opioid intake in group O was 28.2 ± 13.9 days. No permanent pain medication was used for other conditions than LBP. Medial facetectomy was not necessary in any operative case. The mean operative time was 78 ± 32.9 min and did not differ significantly between both groups (O: 76 ± 39 min vs. 83 ± 32 min; *p* > 0.05). Exact blood loss was not documented as it did not exceed 150 ml in any operative case. No blood transfusions were administered in patients included in the study. Two cases of intraoperative adverse events were reported (4%) caused by intraoperative durotomy. No revision surgery was required. None of the patients received a nerve root or facet joint injection postoperatively (Table 3). None of the patients used opioids at the 6-week follow-up or later. No pre- or postoperative differences occurred in thermal or mechanical thresholds (*p* > 0.05). Allodynia did not occur in any of the patients. WUR differed significantly between groups at 1-week follow-up, showing higher values in group NO (O: 1.1 ± 0.32 vs. NO: 2.2 ± 0.79; p = 0.025) (Fig. 1).

**Table 2:** Demographic and clinical characteristics of 52 patients with single lumbar disc herniation

		Group NO, *n* = 35	Group O, *n *= 17	*p* value
Demographic characteristics				
Female/male ratio		16:19	05:12	0.266
Age	In years *(SD)*	42 (± 4)	47 (± 11)	0.183
BMI	In kg/m2 *(SD)*	27 (± 4)	25 (± 2)	0.118
Smoking, *n (%)*		19 (54)	10 (58)	0.195
Cigarettes per day *(SD)*		7 ± 9	7 ± 8	0.936
Alcohol	None, *n (%*)	7 (20)	6 (35)	0.182
	Weekly, *n (%)*	1 (2)	1 (5)	
	Incidentally, *n (%)*	27 (77)	10 (58)	
ASA score	1, *n (%)*	19 (54)	12 (70)	0.266
	2, *n (%)*	16 (45)	5 (29)	
Nerve root injection with steroid, *n (%)*		7 (20)	4 (23)	0.772
Pain characteristics				
Passive leg raising test	Negative, *n (%)*	9 (26)	1 (5)	0.092
	Positive, *n (%)*	26 (74)	16 (94)	
Radicular pain	L3, n (%)	2 (6)	2 (11)	0.192
	L4, *n (%)*	3 (8)	2 (11)	
	L5, *n (%)*	16 (46)	4 (23)	
	S1, *n (%)*	14 (40)	9 (52)	
During of drug intake	In days (*SD)*	27 (± 14)	28 (± 14)	0.881

**Table 3:** Pain medication in the in the opioid group (O) and in the non-opioid group (NO)

	Group NO, *n* = 35		Group O, *n* = 17	
	sum	p.p	sum	p.p
Antiepileptic drugs				
Gabapentin, mg/d	1800	900 (2/35)	1200	1200 (1/17)
Pregabalin, mg/d	300	300 (1/35)	0	0 (0/17)
Non-opioid analgetics				
Naproxen, mg/d	1000	1000 (1/35)	4000	1000 (4/17)
Metamizole, mg/dl	2000	2000 (1/35)	10.500	1750 (6/17)
Paracetamol, mg/d	7500	1875 (4/35)	8000	2000 (4/17)
Diclofenac, mg/d	650	130 (5/35)	100	100 (1/17)
Dexibuprofen, mg/d	5000	1000 (5/35)	4000	1000 (4/17)
Weak opioid analgetics				
Tramadol, mg/d	0	0 (0/35)	1800	180 (10/17)
Strong opioid analgetics		0 (0/35)		
Oxycodone, mg/d	0	0 (0/35)	65	21 (3/17)
Piritramid, mg/d	0	0 (0/35)	49.5	8.2 (6/17)

**Fig. 2:**
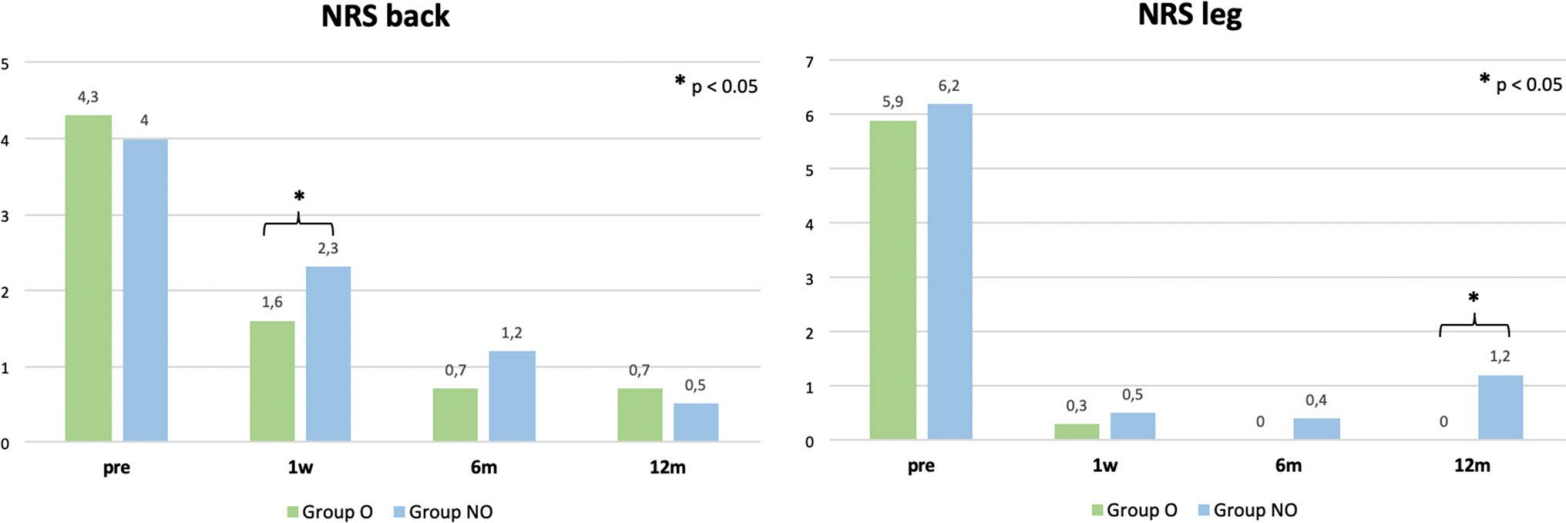
Numeric rating scale (NRS) for back and leg pain among group O and group NO preoperatively, 1 week, 6 months, and 12 months postoperatively

No baseline differences occurred in NRS back or leg (NRS back: O: 4.3 ± 2.6 vs. NO: 4.0 ± 2.7; *p* = 0.841; NRS leg: O: 5.9 ± 2.8 vs. NO: 6.2 ± 2.8; *p* = 0.690). Low back pain on NRS was rated significantly higher in the non-opioid group after 1-week follow-up (O: 1.6 ± 1.5 vs. NO: 2.3 ± 2.3; p = 0.026). Radicular pain revealed to be higher in the NO group after 12 months of follow-up (O: 0.0 ± 0.3 vs. 1.2 ± 2.7; *p* = 0.023) (Fig. 2). The analysis of BDI, COMI, and PD-Q demonstrated no significant intergroup differences pre- and postoperatively (*p* > 0.05). The ODI indicated a trend to greater disability in group O preoperatively (O: 47.6 ± 15.4 vs. NO: 33.6 ± 16.6; *p* = 0.009) but adjusted in the first week after surgery (O: 30.2 ± 15.6 vs. NO: 25.2 ± 17.; *p* = 0.182). Differences were particularly found for pain intensity (O: 3.1 ± 1 vs. NO: 2.2 ± 1; *p* = 0.018), personal care (O: 1.2 ± 0 vs. NO: 0.7 ± 0; *p* = 0.046), sitting (O: 2.7 ± 1 vs. NO: 1.7 ± 1; *p* = 0.013), and travelling (O: 2.8 ± 1 vs. NO: 1.4 ± 1; *p* = 0.002). Additionally, the NO group showed a significantly higher quality of life in EQ-5D preoperatively (O: 0.80 ± 0.07 vs. NO: 0.85 ± 0.07; *p* = 0.029). ODI sum score, COMI, PD-Q, and EQ-5D demonstrated a remarkable increase in the quality of life, overall outcome, and disability 12 months after lumbar sequesterectomy in both groups (*p* < 0.005) (Table 4).

**Table 4:** Questionnaire results: Oswestry disability index (ODI), core outcome measure index (COMI), Beck depression inventory (BDI), painDETECT questionnaire (PD-Q), EuroQol-5Dimension (EQ-5D) preoperatively, 1 week, 6 months, and 12 months postoperatively

Questionnaire	Pre			1w			6 m			12 m			Pre-12 m	
	NO	O	p	NO	O	p	NO	O	p	NO	O	p	NO ∆p	O ∆p
ODI sum	33.0 ± 17	46.2 ± 15	**0.009**	24.0 ± 16	30.2 ± 17	0.214	8.6 ± 10	7.5 ± 8	0.725	8.6 ± 10	7.6 ± 8	0.745	**0.000**	**0.000**
Pain intensity	2.2 ± 1	3.1 ± 1	**0.018**	1.1 ± 0	0.9 ± 0	0.431	0.9 ± 0	0.6 ± 0	0.25	0.8 ± 0	0.5 ± 0	0.472	**0.002**	**0.000**
Pain intensity	0.7 ± 0	1.2 ± 0	**0.046**	0.6 ± 1	0.4 ± 0	0.752	0.1 ± 0	0.0 ± 0	0.691	0.1 ± 0	0.0 ± 0	0.639	**0.006**	**0.002**
Lifting	1.9 ± 1	2.5 ± 1	0.91	2.0 ± 1	3.4 ± 1	0.016	0.7 ± 0	0.6 ± 0	0.713	0.7 ± 0	1.0 ± 1	0.375	**0.000**	**0.000**
Walking	1.1 ± 1	1.8 ± 1	0.141	1.0 ± 1	1.1 ± 1	0.669	0.1 ± 0	0.0 ± 0	0.719	0.2 ± 0	0.1 ± 0	0.74	**0.001**	**0.003**
Sitting	1.7 ± 1	2.7 ± 1	**0.013**	1.6 ± 1	1.8 ± 1	0.501	0.6 ± 0	0.7 ± 1	0.829	0.6 ± 0	0.5 ± 0	0.956	**0.004**	**0.000**
Standing	2.2 ± 1	2.6 ± 1	0.376	1.7 ± 1	1.6 ± 1	0.846	0.5 ± 0	0.6 ± 0	0.611	0.6 ± 0	0.6 ± 0	0.761	**0.000**	**0.001**
Sleeping	1.3 ± 1	1.4 ± 1	0.917	0.8 ± 0	0.5 ± 0	0.104	0.4 ± 0	0.2 ± 0	0.398	0.5 ± 0	0.4 ± 0	0.978	**0.000**	**0.016**
Sex life	1.5 ± 1	1.8 ± 1	0.51	1.2 ± 1	0.8 ± 1	0.481	0.3 ± 0	0.0 ± 0	0.362	0.2 ± 0	0.0 ± 0	0.679	**0.001**	**0.005**
Social life	1.7 ± 1	2.2 ± 1	0.219	0.8 ± 1	0.8 ± 1	0.754	0.2 ± 0	0.3 ± 0	0.627	0.2 ± 0	0.2 ± 0	0.956	**0.000**	**0.003**
Traveling	1.4 ± 1	2.8 ± 1	**0.002**	1.2 ± 1	1.2 ± 1	0.894	0.3 ± 0	0.4 ± 1	0.806	0.2 ± 0	0.1 ± 0	0.72	**0.002**	**0.001**
COMI	6.3 ± 2	6.8 ± 1	0.399	4.6 ± 1	4.5 ± 1	0.803	1.5 ± 1	1.0 ± 1	0.308	1.2 ± 1	0.7 ± 1	0.319	**0.000**	**0.000**
BDI	7.2 ± 5	6.9 ± 5	0.86	4.3 ± 4	4.4 ± 4	0.938	3.3 ± 4	4.5 ± 4	0.429	3.2 ± 3	4.6 ± 7	0.235	**0.003**	**0.231**
PD-Q	18.4 ± 6	16.8 ± 4	0.376	8.7 ± 6	7.4 ± 4	0.465	5.7 ± 5	4.9 ± 4	0.644	5.8 ± 6	5.5 ± 4	0.853	**0.000**	**0.000**
EQ-5D	0.85 ± 0.1	0.8 ± 0.1	**0.029**	0.9 ± 0.1	0.9 ± 0.1	0.364	0.9 ± 0.1	1.0 ± 0.04	0.582	1.0 ± 0.05	1.0 ± 0.04	0.543	**0.000**	**0.001**

Bold entries mark statistically significant results

## Discussion

The present study investigated whether preoperative opioid medication in patients with single lumbar disc herniation positively influences the postoperative outcome in general and detected by quantitative sensory testing specifically. No differences were found in thermal or mechanical thresholds, and allodynia did not occur in any of the patients but pain values for LBP and radicular pain tended to be lower in patients receiving opioids preoperatively. The same was shown for WUR one week postoperatively. Overall, there was a trend to improved outcome in the opioid group.

In our cohort, no differences were found in mechanical or thermal thresholds at any time. This differs from results of previous clinical trials and might be explained by different factors [2, 48, 49]. Sample sizes, as well as patient groups (e.g. chronic pain patients, former addicts, younger patients in the healthy control group), vary substantially. Tested pain syndromes, age, and gender seem to influence the outcome as well [46]. For example, in 71–80% of chronic LBP patients, generalized hypersensitivity was detected in previous trials, which could lead to different findings in QST[3, 9]. Moreover, it is well examined that heat and pressure pain thresholds tend to be lower in women and that elderly patients tend to be less sensitive. Also, this may lead to different reference values [35, 48]. Further factors leading to diverging results might be the heterogeneity of opioid medication, as well as the duration and dose of opioid intake, in studies investigating the influence of these drugs. It is still not fully investigated whether changes in pain sensitivity might become more detectable with prolonged opioid treatment [26].

According to our data, patients not using opioids preoperatively experienced a significantly higher WUR one week after surgery, while at the subsequent follow-ups, no differences between the groups were detected anymore. This might indicate that opioids are able to inhibit wind-up in the short term. WUR is an important tool to examine the processing of nociceptive information in the spinal cord and the central effects of drugs, which are able to modulate the nociceptive system[32]. It is defined as a frequency-dependent increase in the excitability of spinal cord neurons after repetitive stimulation of somatic afferent neurons with stimuli of constant intensity, leading to a summation of these stimuli and production of a more intense discharge [18, 38]. Opioids seem to reduce spinal neuronal responses to afferent C-fibre input and therefore reduce, or even abolish, the generation of wind-up,which has also been shown in opioid-treated patients in the current study [13, 33]. At the same time, wind-up was significantly more prominent in the non-opioid patient group at 1-week follow-up. This finding might be explained by the fact that inflammation and injury lead to a prolonged noxious stimulation. This can enhance the excitability of spinal cord neurons, even in adjacent areas not affected by the inflammation, and might evoke an increase in the degree of wind-up as well as a reduction of the threshold for the induction [18].

Further, prior to the operation, there were differences detected in some subsets of the ODI. Patients who used opioids tended to suffer from more intense pain experienced more disability and less quality of life. These findings are in accordance with other studies [34, 47]. Patients who were preoperatively treated with opioids might have experienced more intense pain over a longer period of time, as chronic pain is well known to have an impact on daily chores, social life, and work and is correlated with low scores for quality of life[41]. Therefore, we assume opioids were prescribed for severely affected patients as a matter of fact. Nonetheless, at the 1-year follow-up, both groups had the same outcome in ODI sum score, COMI, PD-Q, and EQ-5D. However, this may pose a limitation of our non-randomized study, as more severely affected patients may benefit from surgery even more than patients presenting with milder symptoms [50].

While there were no baseline differences found in the NRS, patients receiving non-opioid pain medication preoperatively rated higher NRS on low back pain 1 week after surgery and had a significantly higher disposition to radicular pain 12 months after surgery. These results suggest that preoperative administration of opioids may contribute to postoperative analgesia by blocking the transmission of pain impulses to the central nervous system and thus inhibiting spinal hyperactivity, which results in lower postoperative pain scores [1, 28]. We hereby state that this might be a beneficial effect on patients, who fulfil the criteria for preoperative opioid administration through their severe impairment. The herewith shown data does not allow a clear statement on preventive opioid admission for the value of reduced postoperative pain.

The strength of the current study is the prospective data collection of a homogenous group of patients by using a standardized examination pattern and one single investigator to perform the QST pre- and postoperatively to minimize confounding factors. Preoperative duration of symptoms was comparable between O and NO patients. Limitations of this study are the small patient population and the variable duration of pain medication usage in patients. Even though intraoperative thecal sac retraction was kept to a minimum, long-term impact on radicular pain is not well studied. Further research with a greater number of patients should be implemented.

## Conclusion

Even though worse scores were detected in the opioid group preoperatively, opioids seem to be a positive predictor for the early postoperative pain outcome in patients undergoing lumbar sequesterectomy. This might be especially true for low back pain values one week postoperatively, as well as for radicular pain 1 year after surgery. Findings might indicate diminished neurogenic remodelling by preoperatively used opioids. Nevertheless, the current study includes limitations and further prospective trials are necessary to reach a final decision on opioid treatment in lumbar disc herniation.

## Data Availability

The datasets used and/or analysed during the current study available from the corresponding author on reasonable request.
